# What is the micro- and nanoplastics impact on pathogenic microorganisms?

**DOI:** 10.3389/fmicb.2025.1718190

**Published:** 2025-12-08

**Authors:** Iris Romero-Andrada, Alba Hernández-Bonilla, Jose Domínguez, Alicia Lacoma

**Affiliations:** 1 Institut d'Investigació Germans Trias i Pujol, Badalona, Spain; 2 Departament de Genètica i Microbiologia, Universitat Autònoma de Barcelona, Barcelona, Spain; 3 CIBER de Enfermedades Respiratorias, Instituto de Salud Carlos III, Madrid, Spain

**Keywords:** nanoplastic, microplastic, pathogen, infection, human

## Introduction

Micro- and nanoplastics (MNPLs) are emerging pollutants that have been detected across diverse ecosystems, including the human body. In fact, increasing evidence demonstrates MNPLs accumulation in human tissues, with numerous studies employing diverse analytical methods to detect and characterize them in different human samples (Huang et al., 2022; Jenner et al., 2022; Salvia et al., 2023). Notably, a recent study published in Nature Medicine reported the presence of MNPLs in human liver and brain samples, with an observed association between elevated MNPLs presence in brain samples from individuals diagnosed with dementia (Nihart et al., 2025). While MNPLs potential effects on toxicity are well documented [World Health Organization, 2022; Directorate-General for Environment (European Commission), and University of the West of England (UWE), 2023; Lamoree et al., 2025], their impact on microorganisms remains largely unexplored. In alignment with the perspectives highlighted in two up-to-date publications (Editorial, 2025; Balakrishnan, 2025), and given the widespread presence of MNPLs in ecological and host-associated environments, we intend to share the main findings with regard to MNPLs impact on microorganisms and infection, as well as to pinpoint the gaps and needs in the field from a health-related point of view.

## MNPLs and bacterial communities

The vast majority of studies investigating the interaction between microorganisms and MNPLs, focus on the effects of the latter on environmental microbial communities, mostly from aquatic ecosystems. However, growing body of evidence highlights the detrimental effects of MNPLs on animal microbiomes, pointing out the scarcity of research regarding this subject, and more specifically, on the human microbiome.

For example, the study carried out by Rocabert et al. (2025), investigated the impact of polystyrene and polylactic acid on *Drosophila melanogaster* gut microbiome. Their findings demonstrate that both polymers induce a reduction in the diversity and richness of the fly's microbiome, being the effects of polystyrene greater than those of polylactic acid. Additionally, the *Firmicutes*/*Bacteroidetes* ratio, which is a widely used biomarker in gut microbiota studies, was reduced in all MNPLs treatments, supporting the conclusion that MNPLs induce significant changes in microbial community composition, leading to dysbiosis. Similarly, research done with zebrafish exposed to polystyrene nanoplastics (NPs) up to 28 days, revealed significant gut microbiota dysbiosis, which was characterized by an increase in pathogenic microorganisms from the genera *Pseudomonas, Shewanella, Plesiomonas, Aeromonas*, and *Mycobacterium* (Rehman et al., 2025).

To explore the relevance of these findings to human health, Tamargo et al. (2022) simulated gastrointestinal digestion of polyethylene terephthalate and subsequently exposed human fecal inoculums from two healthy donors. Their results revealed a reduction in total viable bacterial counts and significant shifts in specific microbial populations, consistent with a dysbiotic signature in the colonic microbiota. A recent review by Thin et al. (2025) reveals that upon microplastics (MPs) exposure there is a consistent disruption of human gut microbial communities, associated with alterations of metabolic functions, chronic inflammation, and an enrichment of pathogenic species. These findings confirm the results obtained using animal models, and could also mirror the scenario occurring in the lungs.

Exposure to MNPLs in murine models has been associated with both, gut and lung dysbiosis, suggesting potential implications for the host's health. It has been observed that polystyrene MNPLs are capable of inducing nasal and lung dysbiosis in mice, although the alterations are more severe following exposure to MPs. Some bacterial genera that were enriched upon exposure to MNPLs can be directly related to pathogens or pathogenic processes, such as *Staphylococcus* (airway colonizer and associated with chronic airway diseases), and *Fusobacterium* (exacerbations in chronic obstructive pulmonary disease in mice) (Zha et al., 2023). A study involving C57BL/6 mice that were exposed to polyethylene terephthalate NPs during 12 h a day for 3 months, showed lung microbiota reprograming, with alterations at the phylum and species levels. On the one hand, the relative abundance of *Prevotella* increased, which has a proinflammatory function, as it mediates inflammation of mucosal membranes and promotes systemic dissemination of inflammatory mediators. On the other hand, the abundance of the probiotic *Eubacterium siraeum* decreased; this bacterial species significantly contributes to the promotion of anti-inflammatory molecule secretion (Gu et al., 2025). Thus, not only the lung microbiota is altered upon MNPLs exposure, but the lung microenvironment shifts toward a proinflammatory profile.

Taken together, these results suggest that MNPLs exposure is linked to the decrease of key microbial taxa essential for maintaining the gut and lung microbiomes, as well as for supporting immune homeostasis and preserving organ barrier function, rendering the host more susceptible to suffer infections caused by both primary and opportunistic pathogens.

## MNPLs—microorganisms' interactions

**Figure 1:**
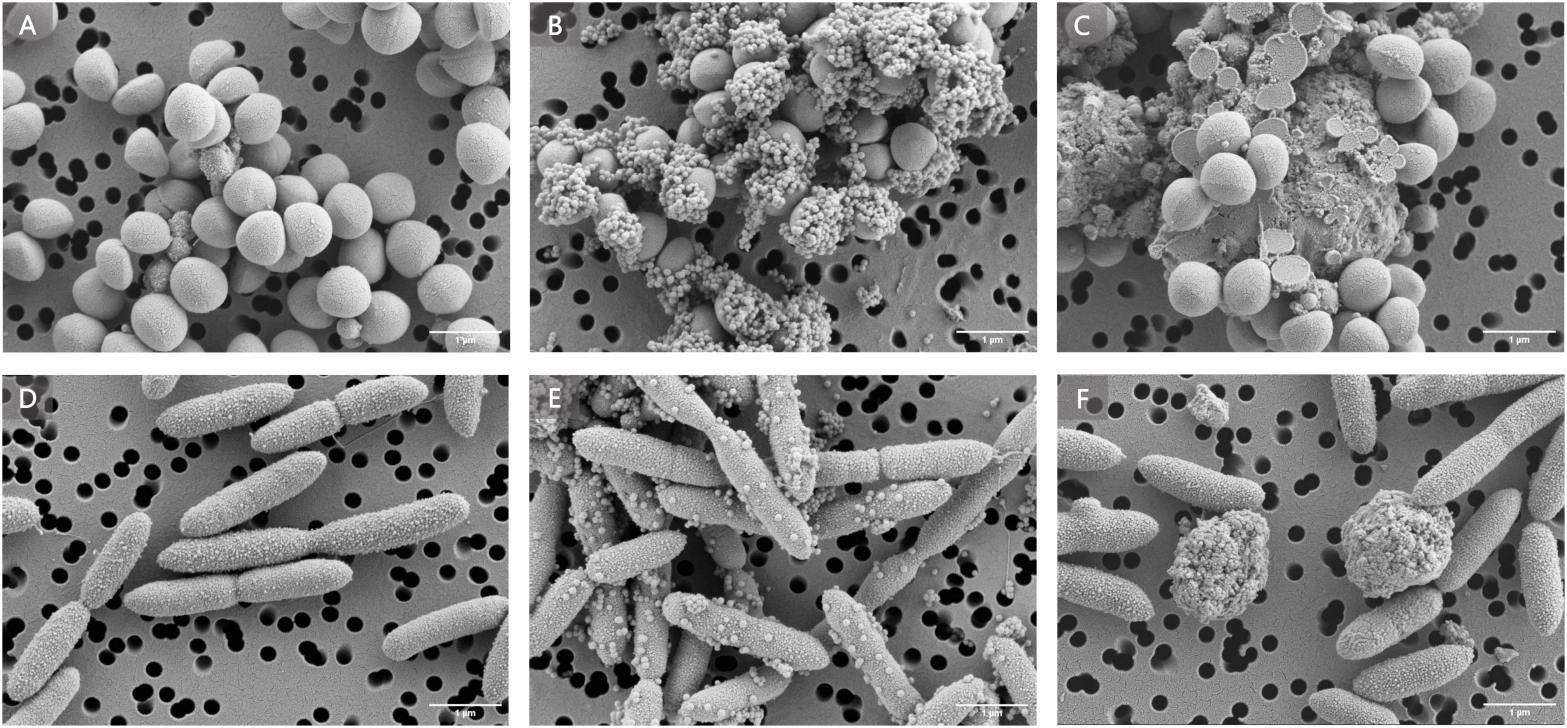
Scanning electron micrographs of *Staphylococcus aureus* and *Pseudomonas aeruginosa* alone (**A** and **D**, respectively), with 150 μg/mL 50 nm polystyrene particles (**B** and **E**, respectively), and with 150 μg/mL 150 nm polyethylene terephthalate particles (**C** and **F**, respectively).

Our knowledge on the interaction between MNPLs and microorganisms is mostly focused on aquatic environments, where bigger sized MPs act as surfaces for microbial colonization, forming the so-called “plastisphere” (Zhai et al., 2023). However, what happens when NPs, smaller in size than most microorganisms, interact with each other? A study published in Scientific Reports (Zajac et al., 2023) has demonstrated that polystyrene NPs attach to the bacterial membranes of the relevant human pathogens *Staphylococcus aureus* and *Klebsiella pneumoniae*. This interaction alters the bacterial surface potential, which could influence their ability to adhere to or invade eukaryotic host cells or evade the immune response mechanisms. Consistent with this study, we have also observed that MNPLs closely interact with *S. aureus* and *Pseudomonas aeruginosa* (Figure 1) (Tytarenko et al., 2023). However, it is important to consider that variations in MNPLs charge and composition, as well as bacterial cell wall structure, may influence the nature of these interactions.

In a study conducted by Gross et al. (2025), the effects of MNPLs concentration, composition, and size were studied on *Escherichia coli* biofilms. They demonstrated that MPs presence increased resistance to ampicillin, ciprofloxacin, doxycycline, and streptomycin. Additionally, all tested polymers (polystyrene, polyethylene, and polypropylene) induced a significantly higher minimum inhibitory concentration (MIC) compared to the MPs-free controls, and for polystyrene, the absolute MIC was higher than the clinical breakpoint of ciprofloxacin for *E. coli*. Furthermore, the presence of MPs selected for better biofilm formers and caused impaired bacterial motility, being the latter also associated with increased biofilm formation. All in all, the study showed a correlation between the MPs presence and an increase in biofilm-associated antimicrobial resistance, which is often linked to recalcitrant infections typically found in healthcare settings. MNPLs can also contribute to the spread of genes related to antimicrobial resistance and virulence, as the plastisphere is a suitable niche for horizontal gene transfer among the different microbial communities that are present (Luo et al., 2023).

Similarly, a study using the pathogenic *E. coli* strain O157:H7 highlights the importance of the NPs surface charge as a physiological stress inducer, mainly affecting bacterial growth and viability. *E. coli* planktonic cells that were exposed to positively and negatively charged NPs, exhibited a significant upregulation of different metabolic pathways that were linked to oxidative stress response, antibiotic resistance, and drug metabolism; additionally, negatively charged NPs upregulated pathogenicity-related pathways involving flagellum-dependent motility and cell localization. In the case of *E. coli* biofilms, the Shiga toxin A subunit gene was upregulated, as well as the adhesion-related gene *eaeA*, which encodes for a protein that plays a crucial role in the *E. coli* attachment to the host's mucosal surface. Several genes involved in biofilm formation were also upregulated, while two stress-response genes were downregulated under all tested conditions, probably due to the protective action of the biofilm exopolysaccharide which was alleviating the cell stress-response (Nath et al., 2025).

A recent investigation employing simulated lung fluids described the impact of different charged polystyrene particles on the commensal *Streptococcus salivarius*, and on the opportunistic pathogen *P. aeruginosa*. In the case of *S. salivarius*, exposure to aminated polystyrene and its aged version caused an increase in oxidative stress, bacterial membrane disruption and overall reduced growth and viability. If this phenomenon is translated to other commensal microorganisms, the overall homeostasis of the respiratory microbiome could be affected, leading to a potential microbiome dysbiosis. Additionally, in the case of *P. aeruginosa*, oxidative stress and bacterial membrane disruption also occurred; however, it also triggered stress-induced virulence, as it was demonstrated by an increase in pyoverdine secretion. Thus, NPs presence in the airways not only could affect the health of our respiratory microbiome, but also increase the virulence of respiratory opportunistic pathogens (Shao et al., 2025).

Despite we have few hints on which might be some of the affected pathogenic pathways, extensive research is still required to elucidate the impact of MNPLs on host-pathogen interactions, pathogenic processes, and virulence traits.

## MNPLs and infection

To date, a handful of articles directly assess the impact of MNPLs on the infectious process itself, either *in vivo* or *in vitro*. Moreover, only two of them employ human pathogens and human cell lines, which once more illustrates the lack of investigation on this specific topic.

For instance, research involving human intestinal epithelial cells and *Galleria mellonella* larvae has demonstrated that when the models were pre-exposed to MNPLs, *Candida albicans* cells exhibited a greater invasive capacity. In fact, in the *in vivo* model, the presence of MNPLs before or in conjunction with *C. albicans*, caused a mortality of the 80% and 100%, respectively, of the *G. mellonella* larvae at 48 h post-infection (Maione et al., 2023). Similarly, honeybees that were fed with polystyrene NPs for 21 days and were later infected with the Israeli acute paralysis virus, exhibited a significant increase in viral copies compared to the group that was not exposed to NPs. A matching trend occurred in terms of honeybee survival (Deng et al., 2021). In our experience, we have observed that exposure of *S. aureus* to polystyrene NPs for 2.5 h enhances its adhesion and infection capacity on a 2D organoid model generated from human embryonic pluripotent stem cells. In fact, intracellular bacterial counts were one order of magnitude higher when *S. aureus* was exposed to NPs compared to the non-exposed bacteria (Romero et al., 2024).

MNPLs often trigger a prolonged immune response, which could lead to chronic immune system activation and energy expenditure, potentially leading to a decreased immune response to infection. This phenomenon has been observed in mussels infected with *Vibrio parahaemolyticus*, where MNPLs exposure compromised their immune response, particularly following long-term exposure (Sun et al., 2024).

A recent study by Edbauer et al. (2025), investigated the inhibitory effect of MNPLs on phagocytosis and intracellular killing using THP-1 human monocytes. On the one hand, upon exposure to polystyrene particles of different sizes and surface modifications, it was observed that *E. coli* phagocytosis decreased in a time and concentration-dependent manner. Additionally, the stimulatory action of lipopolysaccharide on phagocytosis was inhibited by NPs presence. On the other hand, NPs inhibited THP-1 intracellular killing of *E. coli* in a concentration dependent manner.

## Conclusion

Considering the growing body of evidence indicating the detrimental effect of MNPLs on human health, a direct causal link to human pathologies has yet to be firmly established. It remains imperative to explore the potential MNPLs role in disease, particularly in chronic conditions that could be linked to infection and those associated with occupational exposure.

It is also crucial to further investigate MNPLs impact in microbial pathogenesis in terms of genotypical (mutations and alterations in gene regulation) and phenotypical (growth kinetics, cell wall composition, metabolism, drug resistance and virulence) traits. The clinical outcome of an infectious disease is driven by the interactions between host, microbial and environmental factors. However, the potential role of MNPLs in such scenario is still in its infancy. Following the same train of thought, immune response to infection might also be affected by MNPLs, altering phagocyte and non-phagocyte cell activity, as well as cytokine and immunomodulatory mediator secretion.

The experimental approaches to explore these interactions are diverse and of different complexity. However, all of them are subject to the same constraint, given the virtually unlimited diversity of plastic polymers, sizes, shapes, chemical modifications, and additives. Another critical factor is exposure time, being most studies focused on short-term exposure; however, longer exposure times would better mimic the real scenario. Furthermore, we also lack “true to life” concentrations, as the methods for MNPLs quantification in different environments (air, soil, and water) and matrices are not fully standardized. This huge variability hinders protocol optimization, hampering the comparison between published studies and the harmonization required for future risk assessment.

In fact, this field of research has already caught the attention of the European Commission as it is outlined in the European Union Horizon 2025 call, under the research line on “Advancing knowledge on the impacts of micro- and nanoplastics on human health.” We anticipate that this research field will experience significant growth in the coming years, driven by increasing public awareness of pollution and infectious diseases, particularly in the aftermath of the COVID-19 pandemic.
